# Clinical evaluation of antiseptic mouth rinses to reduce salivary load of SARS-CoV-2

**DOI:** 10.1038/s41598-021-03461-y

**Published:** 2021-12-22

**Authors:** Maria D. Ferrer, Álvaro Sánchez Barrueco, Yolanda Martinez-Beneyto, María V. Mateos-Moreno, Verónica Ausina-Márquez, Elisa García-Vázquez, Miguel Puche-Torres, Maria J. Forner Giner, Alfonso Campos González, Jessica M. Santillán Coello, Ignacio Alcalá Rueda, José M. Villacampa Aubá, Carlos Cenjor Español, Ana López Velasco, Diego Santolaya Abad, Sandra García-Esteban, Alejandro Artacho, Xavier López-Labrador, Alex Mira

**Affiliations:** 1 Department of Health and Genomics, FISABIO Foundation, Avda. Cataluña 21, 46020 Valencia, Spain; 2 ENT and Cervicofacial Surgery Department, Fundación Jiménez Díaz University Hospital, Madrid, Spain; 3 Villalba General University Hospital, Madrid, Spain; 4 Department of Dermatology, Stomatology and Radiology, Faculty of Medicine and Dentistry, University of Murcia, Murcia, Spain; 5 CIBER Epidemiología y Salud Pública, Madrid, Spain; 6 Oral and Maxillofacial Department, Hospital Clínico Universitario de Valencia, Valencia, Spain; 7 Department of Dental Clinical Specialties. School of Dentistry, Madrid Complutense University, Madrid, Spain; 8 Infectious Diseases Unit, Hospital Clínico Universitario Virgen de la Arrixaca, IMIB, Murcia, Spain; 9 Department of Dentistry, European University of Valencia, Valencia, Spain

**Keywords:** Randomized controlled trials, Viral infection

## Abstract

Most public health measures to contain the COVID-19 pandemic are based on preventing the pathogen spread, and the use of oral antiseptics has been proposed as a strategy to reduce transmission risk. The aim of this manuscript is to test the efficacy of mouthwashes to reduce salivary viral load in vivo. This is a multi-centre, blinded, parallel-group, placebo-controlled randomised clinical trial that tests the effect of four mouthwashes (cetylpyridinium chloride, chlorhexidine, povidone-iodine and hydrogen peroxide) in SARS-CoV-2 salivary load measured by qPCR at baseline and 30, 60 and 120 min after the mouthrinse. A fifth group of patients used distilled water mouthrinse as a control. Eighty-four participants were recruited and divided into 12–15 per group. There were no statistically significant changes in salivary viral load after the use of the different mouthwashes. Although oral antiseptics have shown virucidal effects in vitro, our data show that salivary viral load in COVID-19 patients was not affected by the tested treatments. This could reflect that those mouthwashes are not effective in vivo, or that viral particles are not infective but viral RNA is still detected by PCR. Viral infectivity studies after the use of mouthwashes are therefore required. (https://clinicaltrials.gov/ct2/show/NCT04707742; Identifier: NCT04707742)

## Introduction

The coronavirus disease 2019 (COVID-19) outbreak was quickly declared by the World Health Organization (WHO) a public health emergency of international concern and has given rise to one of the most dramatic pandemics in recent human history^1^. The disease is caused by the Severe Acute Respiratory Syndrome Coronavirus 2 (SARS-CoV-2) virus, a single-stranded enveloped RNA virus which belongs to the *betacoronavirus* genus from the *Coronaviridae* family^2^.

Because no effective treatment for COVID-19 is currently available, most public health measures to contain the pandemic are based on preventing the spread of the pathogen. The virus is transmitted by the respiratory route (respiratory droplets and aerosols) and by direct contact with contaminated surfaces and subsequent contact with nasal, oral or ocular mucosa^3^. Although patients with symptomatic COVID-19 have been the main source of transmission, asymptomatic and pre-symptomatic patients also have the ability to transmit SARS-CoV-2^4^. Higher viral loads are detected after the onset of COVID-19 symptoms, being significantly higher in the nose compared to the throat^5^.

Angiotensin-converting Enzyme 2 (ACE2) is the main cellular receptor for SARS-CoV-2, which interacts with the spike protein to facilitate its entry. ACE2 receptors are highly expressed in the oral cavity and present at high levels in oral epithelial cells^6^. The mean expression of ACE2 was higher in the tongue compared to that in other oral tissues and it has been found to be higher in the minor salivary glands than in the lungs^6^. These findings strongly suggest that the oral cavity and specifically the saliva may be a high-risk route for SARS-CoV-2 infection^7^. Thus, strategies reducing salivary viral load could contribute to reduce the risk of transmission. Furthermore, studies using macaques as animal models have shown that SARS-CoV-1 (which has an 82% genomic similarity with SARS-CoV-2) persists for 2 days in oral mucous membranes before its diffusion to the lower respiratory tract. This offers an interesting preventive and therapeutic window of opportunity for the control of this disease^8^. For this reason, the use of mouthwashes with antiseptics that have virucidal activity can be a simple preventive strategy that could easily be applied in the general population.

It is widely known that antimicrobial mouthwashes reduce the levels of oral microorganisms including bacteria and fungi, and there are promising in vitro data and clinical studies showing virucidal effects against influenza^9^, murine norovirus or herpes simplex virus^10,11^, among others. In relation to coronaviruses, there was also in vitro evidence that povidone-iodine, a common active ingredient in some oral antiseptics, has efficient virucidal activity against Middle East Respiratory Syndrome Coronavirus (MERS-CoV) and SARS-CoV-1^12,13^. Stimulated by these background, numerous research teams have evaluated the in vitro efficacy of different oral antiseptics against SARS-CoV-2 and other coronaviruses, showing potent virucidal activity of chlorhexidine (CHX), cetylpyridinium chloride (CPC), povidone-iodine (PVP-I), essential oils or hydrogen peroxide (H_2_O_2_)^14–18^. However, there is very limited clinical evidence for the effectiveness of mouth rinses against SARS-CoV-2 in vivo and there is an urgent need to obtain reliable data in placebo-controlled randomized clinical trials where the comparative effect of different oral antiseptics can be experimentally verified^19^.

Thus, in the current manuscript we present the results of a multi-centre, blind, parallel-group, placebo-controlled randomised clinical trial testing the effect of four different mouthwashes (CPC, CHX, PVP-I and H_2_O_2_) in the salivary load of SARS-CoV-2 as measured by quantitative real-time PCR at three different timepoints. Our study aims to test whether any of these standard oral antiseptics appear to diminish viral load in saliva and could therefore be used as a strategy to reduce transmission risk in clinical and social settings.

## Material and methods

### Study design

To evaluate the effect of several antiseptics to neutralize or reduce the viral load in vivo of SARS-CoV-2 in saliva samples, a multicentre, randomized, double-blind, five-parallel-group, placebo-controlled trial was designed (ClinicalTrials.gov Identifier: NCT04707742). The present study was approved on 2020/05/8th by the Ethical Committee of the Fundación Jiménez Díaz University Hospital with code EO095-20_FJD-HGV-HIE and all research was performed in accordance with relevant guidelines/regulations.

The study was performed in Madrid, Valencia and Murcia regions in Spain, in five different hospitals: Fundación Jiménez Díaz University Hospital (Madrid, Spain), Villalba University General Hospital (Madrid, Spain), Infanta Elena University Hospital (Madrid, Spain), Virgen de la Arrixaca University Hospital (Murcia, Spain) and Clínico de Valencia University Hospital (Valencia, Spain).

### Patients

Every patient included was previously diagnosed and hospitalized because of SARS-COV-2 infection, being admitted mainly for respiratory pathology. All were adults (age > 18 years) and provided their voluntary written or oral consent to participation according to the ethics committee recommendations. The inclusion criteria included a positive result for SARS-COV-2 RT-PCR test of a respiratory sample in the previous 7 days and the ability to donate saliva samples and perform a mouthwash. Exclusion criteria included patient participation in a COVID-19 research study using experimental drugs, the use of an antiseptic mouthwash for 48 h before the start of the study and any known hypersensitivity or allergy to components of the mouthwashes.

### Randomisation and masking

After approval of consent, the hospital staff responsible for the interventions consecutively assigned each participant a code following the order from a previously randomly generated table provided by other member of the research team. The code consisted of a patient number and a letter corresponding to one of the five study groups (A, B, C, D and E), that were unknown to the personnel who performed RNA extraction and qPCR and to those that analyzed the data. In this way, participants were randomly assigned to one of the five treatment groups and the blind for the patients was achieved by using identical tubes with the same volume for both mouthwashes and placebo. Patients receiving antiviral drugs (n = 6, all taking remdesivir) were randomly assigned to the different groups (three patients to the H_2_O_2_ group, one to the PVP-I group and two to the control group).

### Procedures

Every included patient was asked not to eat, drink anything but water, chew gum, smoke or brush their teeth for 1 h prior to sample collection. In addition, it was not allowed to drink for half an hour after the mouthwash and eat for the entire test. For standardization, participants were instructed how to perform the mouthwash and how to donate the sample.

Five mouthwashes were randomized: 2% povidone-iodine (group A), 1% hydrogen peroxide (group B), 0.07% cetylpyridinium chloride (group C), 0.12% chlorhexidine (group D), and distilled water (group E), as the control group. Group C (Vitis Xtra Forte©) and D (Clorhexidina Dental PHB©) rinses were ready to use in their commercial formulas. In the case of the mouthwashes A (Betadine^®^ Bucal 100 mg/mL) and B (Oximen©), the concentrations were adjusted to those indicated by the manufacturer, by diluting commercial formulas with distilled water minutes before rinsing (3 mL of povidone-iodine 10% for oral use with 12 mL of distilled water, in group A; and 5 mL of hydrogen peroxide 3%—with 10 mL of distilled water, in group B).

**Figure 1:**
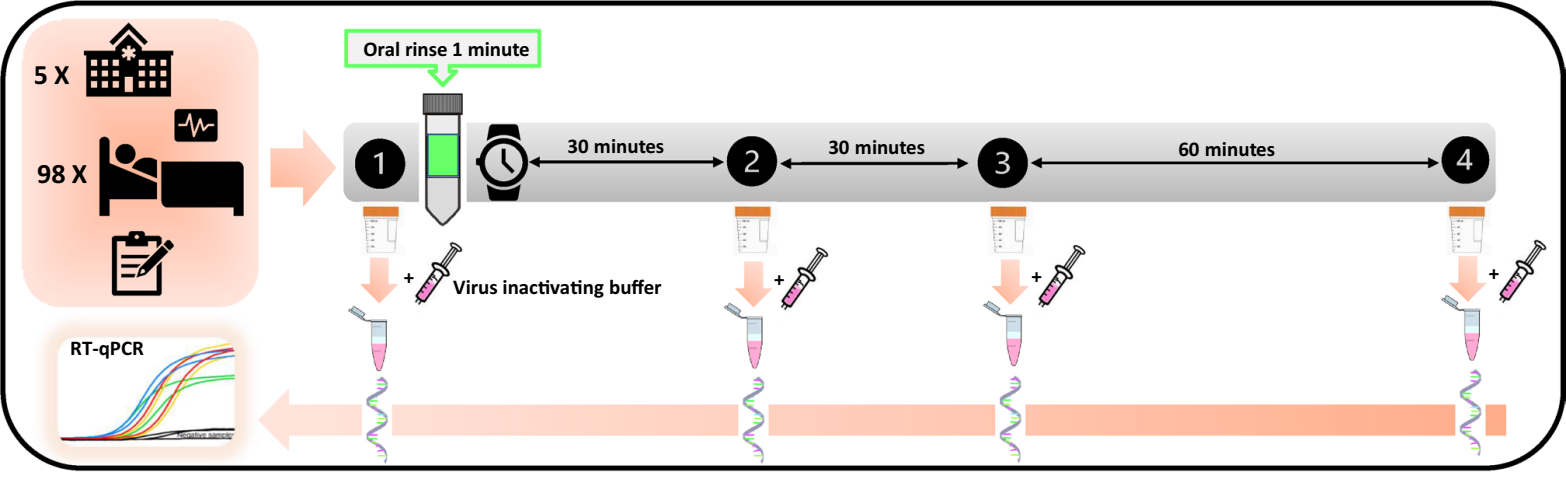
Diagram of the experimental study protocol. The upper left panel illustrates the multicentre study with five hospitals involved, the number of enrolled patients diagnosed and hospitalized because of SARS-COV-2 infection and the record of clinical variables. The sampling of the four non stimulated saliva samples for each patient is represented by the numbers 1, 2, 3 and 4 (corresponding to one basal and three post-mouthwash samples, at 30 min, at 60 min and at 120 min, respectively), as well as the rinsing time for 1 min. In this blind study, participants were randomly assigned to one of the five parallel group: PVP-I (group A), H_2_O_2_ (group B), CPC (group C), CHX (group D) and distilled water (group E, control group). After each sampling, 0.5 mL of saliva was transferred to a sterile Eppendorf tube with 1.5 mL of virus inactivating buffer, illustrated in the figure with a syringe. All samples were taken to the laboratory where RNA extraction and RT-qPCR (reverse transcription—quantitative polymerase chain reaction) was performed.

A total of 4 non stimulated saliva samples were taken for each patient in the morning: one basal and three after a 1-min mouthwash, specifically at 30, 60 min and 120 min. Figure 1 shows a scheme of the study design. The patients were asked to provide each unstimulated saliva sample into a sterile plastic container (at least 0.5 mL of sample) by drooling, avoiding spilling secretions of bronchial origin. Immediately after each sampling, 0.5 mL of saliva was transferred to a sterile Eppendorf tube with 1.5 mL of virus inactivating buffer (60% w/v Guanidine thiocyanate, 50% (v/v) 0.1 M Tris HCl, 11% v/v 0.2 M EDTA pH 8 and 1.3% v/v Triton X-100) (Crick Institute Standard Operation Procedures, accessed May 16th 2020) labeled with the patient code and sample time and kept at 4 °C. The four Eppendorf tubes per patient were introduced in an airtight bag, inside which there was protecting absorbent material in case of undesired opening or breaking. The secondary containers were introduced in a rigid box according to UN3733 standards and sent to a centralized laboratory (FISABIO) by courier service for analysis.

### Nucleic acid extraction and real-time RT-PCR

An aliquot of 1 mL of each saliva sample diluted in the virus inactivating buffer in proportion 1:3, was used for RNA extraction using a standard TRIzol™ reagent (Invitrogen) method^20^.

An assay for SARS-CoV-2 was developed based in the WHO-Charité and U.S. CDC assays, to detect the SARS-CoV-2 E-gene in a multiplex assay together with the human cellular control RNAse-P to ensure reliability of sample collection and nucleic acid extraction before RT-PCR assays^21,22^. SARS-CoV-2 E-gene (FAM) and the housekeeping human RNP gene (Cy5) primer and probe sets were mixed with the qScript XLT One-Step RT-qPCR ToughMix (Quanta BioSciences, USA) and 8 μL of extracted RNA, as per manufacturer instructions. Real-time RT-PCR was performed in a Lightcycler 480II apparatus (Roche Diagnostics, Spain). Only those samples with RNAse-P *Ct* values < 35 were considered valid. The limit of detection of the assay was established to 35 copies using the SARS-CoV-2 E-gene as reference material, either as an insert in a pCDNA plasmid (a gift from Dr. Luis Enjuanes laboratory, CNB-CSIC, Spain) or as encapsulated RNA (a gift from Charité Medical Center, Berlin, though the European Virus Archive program). All samples were run in two replicates, together with previously known positive and negative controls. Virus copies were quantified using a tenfold dilution series standard curve of synthetic RNA (SARS-CoV-2 Standard, Exact Diagnostics, USA). Virus copies were normalized by mL of saliva.

### Statistical analysis

Considering that each volunteer act as their own control, when comparing the viral load values in saliva at every time with respect to the levels prior to rinsing with the mouthwash, a sample size of 15 patients per branch was considered sufficient to identify significant differences between groups of more than 20%, assuming a 10% loss of patients due to abandonment or low viral load and adopting an alpha of 0.05 and a power of 0.8. As 5 branches were programmed in the trial (CPC, CHX, PVP-I, H_2_O_2_ and the control), 75 patients were the minimum number of individuals to be recruited, who were distributed among the different hospitals.

A Wilcoxon signed-rank test was used to test average differences, both for the paired case, when comparing observations in different times for the same individuals, and for the un-paired case, when comparing different treatments based on relative increments of viral load. Also, some tests for association between paired samples using Spearman's correlation coefficient were performed to assess relationship between viral load and other clinical continuous variables. Finally, to evaluate associations between clinical categorical variables and the frequency of responders/non-responders to the treatments, we carried out chi-squared contingency table tests. We define as responder an individual exhibiting an improvement equal to or greater than 90% of basal viral load. All computations and tests were performed using R environment for statistical computing version 3.6.3 and its 'stats' package^23^.

## Results

**Figure 2:**
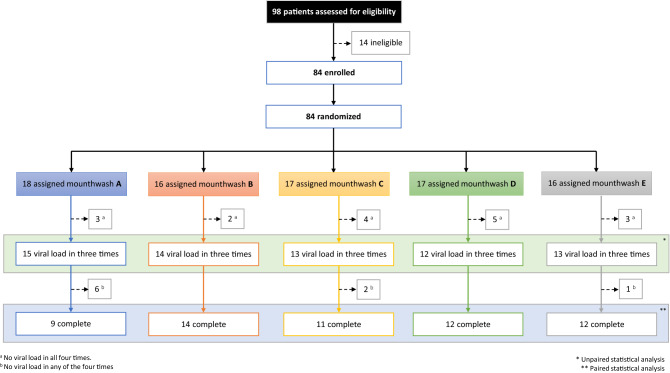
Trial profile. Boxes display the number of individuals recruited for the clinical study and those assigned to each treatment, indicating the number of samples discarded due to undetectable viral load. ^a^No viral load detected at all four times. ^b^No viral load detected in any one of the four sampling times. *Unpaired statistical analysis (three samples per patient available, including basal values). **Paired statistical analysis (all four samples available).

From the 98 individuals screened, 14 did not fulfil inclusion criteria due to COVID-19 diagnostic other than PCR or to a period of more than 7 days since the PCR. The 84 individuals enrolled were randomly assigned to one of the 5 study groups (Fig. 2). After RNA extraction, a control qPCR for the human ribonuclease P gene was performed for all 336 samples, from which 17 produced no result and were discarded. A sample size of 12–15 individuals per group were analysed with 3–4 valid samples (total number of patients: 67), and for paired statistical comparisons, a sample size of 9–14 individuals per group (total number: 58) was used for which all four samples produced valid results.

The mean age of recruited patients was between 54–55 for the five groups with a range of 19–87 years (Table 1). There was a higher proportion of males than females in all five groups. Before the mouthwash, basal viral loads for most patients ranged between 10^2^–10^6^ copies/mL of saliva, and a maximum load of 10^10^ copies/mL was detected. There was no statistically significant correlation in basal viral load with the number of days elapsed since the original diagnostic RT-PCR (Spearman’s correlation coefficient between viral load and days since PCR was − 0.014) or since the day of first symptoms (Spearman’s correlation coefficient − 0.064). There were no statistically significant differences in basal viral load between sexes, nor between patients receiving antiviral drugs (n = 6, all of them taking Remdesivir) and those that did not (n = 61). A trend towards higher basal viral load values in older patients was detected (Spearman’s correlation coefficient 0.24, p-value = 0.067; Supplementary Fig. 1).

**Table 1:** Clinical variables registered from the patients included in the study. *Days from the positive SARS-COV-2 PCR test (nasopharyngeal swab) performed at hospital. **Days since the appearance of symptoms related with SARS-COV-2 infection.

	A Povidone-iodine (PVP-I)	B Hydrogen peroxide (H_2_O_2_)	C Cetylpyridinium chloride (CPC)	D Chlorhexidine (CHX)	E Control group (control)
**Age (years)**	54 (75–34)	55 (87–33)	54 (81–20)	55 (81–19)	55 (77–33)
0–40	4 (27%)	1 (7%)	3 (23%)	4 (33%)	3 (23%)
41–60	3 (20%)	8 (57%)	4 (31%)	5 (42%)	5 (38.5%)
> 60	8 (53%)	5 (36%)	6 (46%)	3 (25%)	5 (38.5%)
**Sex**					
Female	6 (40%)	6 (43%)	2 (15%)	5 (42%)	3 (23%)
Male	9 (60%)	8 (57%)	11 (85%)	7 (58%)	10 (77%)
PCR positive* (days)					
0–2	9 (60%)	7 (50%)	8 (62%)	5 (42%)	6 (46%)
3–5	2 (13%)	4 (29%)	3 (23%)	5 (42%)	5 (39%)
> 5	4 (27%)	3 (21%)	2 (15%)	2 (16%)	2 (15%)
Symptoms** **(days)**					
0–6	7 (47%)	7 (50%)	3 (23%)	6 (50%)	6 (46%)
7–9	5 (33%)	5 (36%)	9 (69%)	5 (42%)	4 (31%)
> 9	3 (20%)	2 (14%)	1 (8%)	1 (8%)	3 (23%)

**Figure 3:**
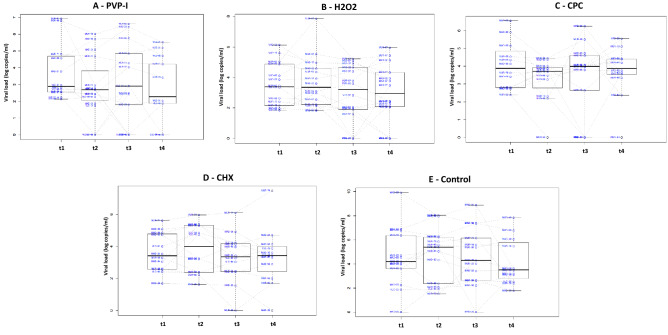
Salivary viral loads for all five treatment groups through time. Box plots show viral load levels, as measured by qPCR, for each of the patients in the group A—PVP-I (povidone-iodine), B—Hydrogen peroxide (H_2_O_2_), C—CPC (cetylpyridinium chloride), D—CHX (chlorhexidine) and E—Distilled water (placebo group) at the four different timepoints (1 for basal and 2, 3 and 4 for the three times after the mouthwash, corresponding to 30, 60 and 120 min after the mouthwash, respectively). Lines joining the samples from the same patient are also shown.

**Figure 4:**
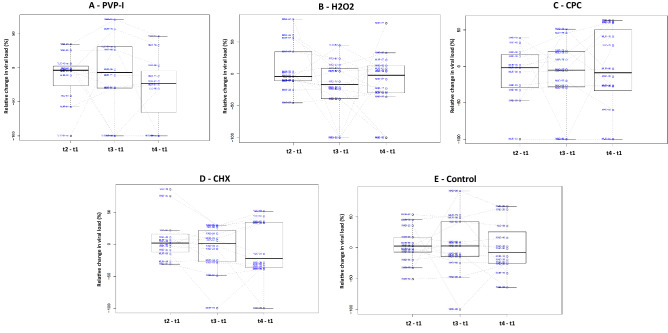
Relative changes in salivary viral load for all five treatment groups after the mouthwash. Box plots show changes of viral load (%) for each of the patients in the group A—PVP-I (povidone-iodine), B—H_2_O_2_, C—CPC (cetylpyridinium chloride), D—CHX (chlorhexidine) and E (placebo group) at the three different times after the oral rinse treatment, relative to the baseline viral load prior to the mouthwash (changes at 30 min: t2–t1; changes at 60 min: t3–t1; changes at 120 min: t4–t1). Lines joining the points from the same patient through time are also shown.

**Figure 5:**
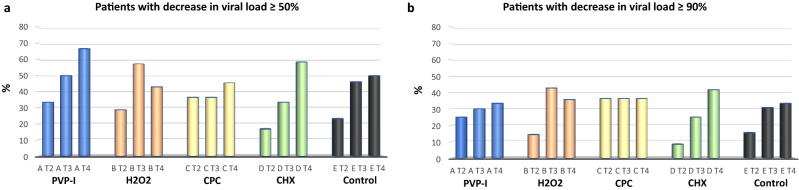
Patients with improvement in viral load values. Bar plots show the percentage of individuals lowering salivary viral load by 50% or higher (**a**), or 90% or higher (**b**) relative to baseline values prior to the oral rinsing treatment, at different times after the mouthwash (t2: 30 min, t3: 60 min and t4: 120 min) in each study group: PVP-I (povidone-iodine), H_2_O_2_, CPC (cetylpyridinium chloride), CHX (chlorhexidine) and control group, respectively.

Viral loads in saliva for all five groups through time are shown in Fig. 3 (individual data for each patient are provided in Supplementary Table 1). None of the tested mouthwashes significantly reduced viral load at any timepoint compared with baseline. However, individual responses were highly divergent, with clear decreases in viral load in some individuals or at some time points and increases at other times. When looking at the relative changes compared to the values before the mouthwash, the maximum effects on viral load were observed 2 h after treatment in PVP-I and CPC groups, with mean viral load reductions around 30% (Fig. 4). The number of individuals that underwent a 50% decrease in viral load increased with the time elapsed since the mouthwash in the PVP-I and CPC groups, reaching 65% and 59% of participants, respectively (Fig. 5a). In the H_2_O_2_ group, the largest effect was seen 1 h after treatment, whereas in the CHX group the effect was seen already at 30 min and was maintained with time. A similar pattern was observed for individuals undergoing a 90% decrease in viral load (Fig. 5b). The advantage of using an antiseptic mouthwash was limited when compared with the distilled water control, where a significant number of patients also underwent a decrease in viral load.

Given the high inter-individual variability in the response, the potential effect of clinical features on treatment efficacy was evaluated. Only a trend was observed for viral load decrease (higher than one order of magnitude) in patients in the PVP-I group 2 h post-treatment when considering individuals for which saliva was collected less than 6 days after first symptoms appeared (p = 0.06, Wilcox test). The effect of oral mouthwashes on salivary viral load remained non-significant when considering only samples with a high or low basal viral load.

## Discussion

The data obtained in the current clinical study indicate that SARS-CoV-2 salivary load, as measured by real-time RT-PCR, was not significantly affected by the use of four widely used mouthwashes. This contrasts dramatically with previous in vitro studies where short exposure times (15–60 s.) of SARS-CoV-2 or other coronaviruses to different oral antiseptics resulted in strong virucidal effects ranging from one to four orders of magnitude^15,16,24,25^. These studies were performed using direct contact of the virus with antiseptics prior to adsorption and infection essays using Vero E6 or Huh7 cell lines. In some cases, addition of compounds like mucin, BSA, fetal calf serum or yeast extract was performed to mimic oral secretions or the naso/oropharynx. However, our data suggest that artificial conditions used to test mouthwashes in vitro may not be extrapolated to the actual effect of those antiseptics in the human oral cavity. For example, salivary glycoproteins are likely to interfere with the antiseptics’ antimicrobial activity, and compounds introduced in the oral cavity have been found to undergo a 1:4 dilution due to the presence of saliva^26^, potentially reducing their effect. In addition, all oral antiseptics tested in the current study have been previously shown to have activity against other microorganisms, including bacteria and/or fungi. Thus, a lower product availability in vivo is expected as a consequence of the antiseptic binding to other microorganisms in the oral cavity.

When present, the virucidal effects observed did not always match the temporal expected pattern based on their substantivity (i.e. their ability to persist in the oral cavity for a period of time). Hydrogen peroxide, for example, is expected to have an immediate effect but its activity may not remain over time due to chemical decomposition. However, the proportion of individuals with a large (> 1 log) reduction in viral load in this group at 1 and 2 h doubled that observed after 30 min of the mouthwash (Fig. 5). CPC, on the other hand, has a high expected substantivity (between 4–6 h), but a 30% of patients with a decreased viral load relative to the control group was already observed 30 min after the mouthwash. Results with CHX did match its expected persistence in the oral cavity, as it increased its efficacy through time. It must be considered that oral clearance of food or drinks due to saliva swallowing can take up to half an hour for a person with a healthy salivary flow. Thus, a potential increase in viral load after the mouth rinse could be expected if viral particles are released from oral mucosa and tongue tissues, but our data did not show this potential increase at the 30-min sample. Future studies should therefore also focus on determining SARS-CoV-2 levels during the first 30 min after the mouthwash, in order to determine the initial dynamics of viral load after the use of oral antiseptics. In the current manuscript, a higher proportion of patients appeared to show a decrease in viral load with time, especially in the CHX group, suggesting an initial virucidal effect in those individuals, after which viral particles would be swallowed and not replaced by new viruses.

The data presented in the current study reveal a large inter-individual variation in the observed response. This high variability can partly be due to age differences among subjects, as a trend was found in higher basal viral load in older individuals. The use of anti-viral treatments could of course affect the response, but this effect is probably limited in our dataset because only six out of 84 individuals were under antiviral drug treatment. In addition, although specific instructions were given to participants on how to perform the mouthwash, we cannot discard that the strength or intensity of the procedure was different for different individuals and that this mouthwash performance influenced inter-individual variability in treatment response. Apart from methodological effects, the temporal variability in viral load, which was also observed in the control group, could be influenced by naturally occurring changes in the shedding of viruses from other body niches like the nasopharynx^27^.

The randomized assignment to the different treatments produced groups that did not vary in age or sex, which are factors known to affect disease severity, thereby facilitating inter-group comparisons. Interestingly, a considerable number of patients underwent reduced viral loads through time in the control group, highlighting the importance of a placebo branch to extract valid conclusions. In three of the four previous clinical studies, for example, no control group was included when testing the effect of chlorhexidine (n = 2^28^), H_2_O_2_ (n = 12^29^) or povidone-iodine (n = 4^30^) and the effect observed could have therefore been taking place also in the absence of a mouthwash. In a fourth clinical study to test the effect of three oral mouthwashes (n = 6 on each treatment group), a control branch was included with only two individuals, which happened to increase viral load after the mouth rinse^31^. Thus, when the changes in viral load after the mouthwashes were compared to those observed in the control group, a small relative benefit could be deduced, but the viral load in all individuals when compared to their corresponding basal values did not change after the mouthwash. In our dataset, up to half of the patients in the control group decreased their viral load by 50% after 2 h (Fig. 5), underlining the importance of a control branch with an equivalent sample size to the other branches. In addition, the lack of efficient antiviral drugs that could serve as a positive control in our study limits the evaluation of mouthwashes. This may change in the near future as a continuous, unprecedented effort is being performed aiming to develop efficient treatments against SARS-CoV-2, for example by drug repositioning^32^. Thus, although these drugs are mostly envisaged for systemic treatment, some may be suitable for topic application through mouthwashes.

Although our data reveal that oral mouthwashes have no significant effect on SARS-CoV-2 viral load as measured by qPCR, the possibility remains that those viral particles detected after the treatment could be non-viable. This was proposed by Gottsauner and collaborators in their study with a H_2_O_2_ mouthwash but unfortunately, viral cultures in their samples could only be obtained from one baseline sample in one of the patients, and this hypothesis could not therefore be tested^29^. The possibility of a mouthwash effect on viral infectivity would explain why the virucidal activity appears to increase with time even in the H_2_O_2_ treatment, which is a compound without a prolonged effect, as in the absence of viral colonization from the nasopharynx, oral viral replication would be diminished. Thus, there is an urgent need of doing viral culture experiments with saliva samples collected after mouthwash use to confirm viral viability. Although RNA is a highly unstable molecule and therefore its integrity in saliva is expected to be quickly compromised, the possibility that the qPCR protocol could be detecting RNA from dead viral particles is supported by the fact that persistent viral amplification by PCR has been documented in upper respiratory or fecal samples weeks after the COVID-19 symptoms have disappeared^33,34^.

The potent virucidal effects against different coronavirus detected in vitro resulted in oral mouthwashes being recommended during the pandemic and that they are routinely used around the world in different clinical situations and before dental procedures to reduce infection risk by SARS-CoV-2^1,19^. However, if the action of oral antiseptics in vivo is limited, their use could provide a false sense of security. Thus, we recommend that strict preventive measures should stand in place in clinical settings until viral infectivity studies in cell lines with post-mouthwash saliva are performed. These viral culture studies with oral samples after the use of oral mouthwashes are warranted.

## Supplementary Information

Supplementary Figure 1.

Supplementary Table 1.

Supplementary Legends.

## Data Availability

Clinical data has been previously published in the clinical trial database (https://clinicaltrials.gov/ct2/show/NCT04707742; Identifier: NCT04707742). Additional data are available from the authors upon request.
