# Supplementary figures and images for: Clinical evaluation of antiseptic mouth rinses to reduce salivary load of SARS-CoV-2
Source: Sci Rep. 2021 Dec 22;11:24392. doi: 10.1038/s41598-021-03461-y (PMC8695582; doi:10.1038/s41598-021-03461-y)

Supplementary Figure 1

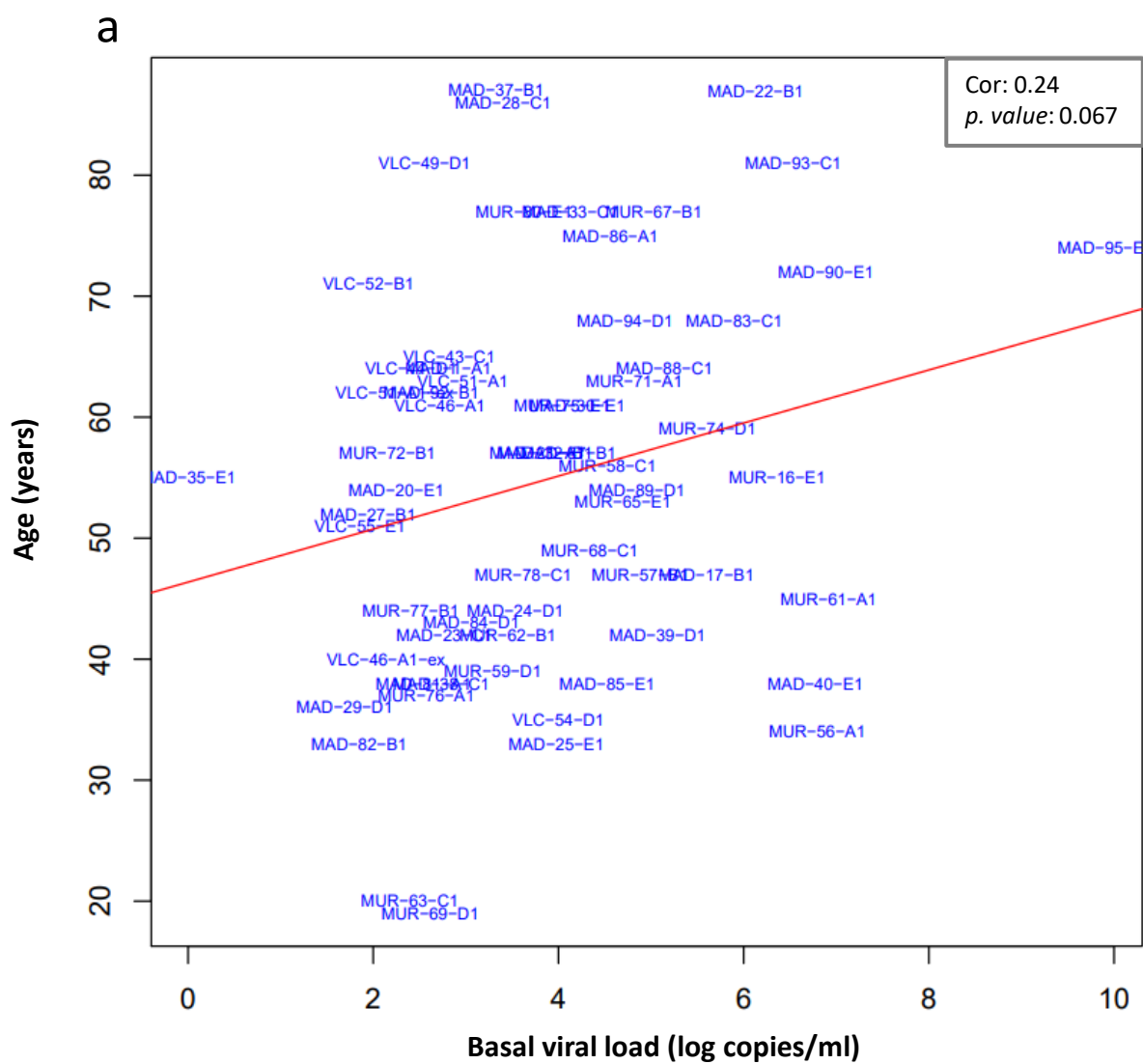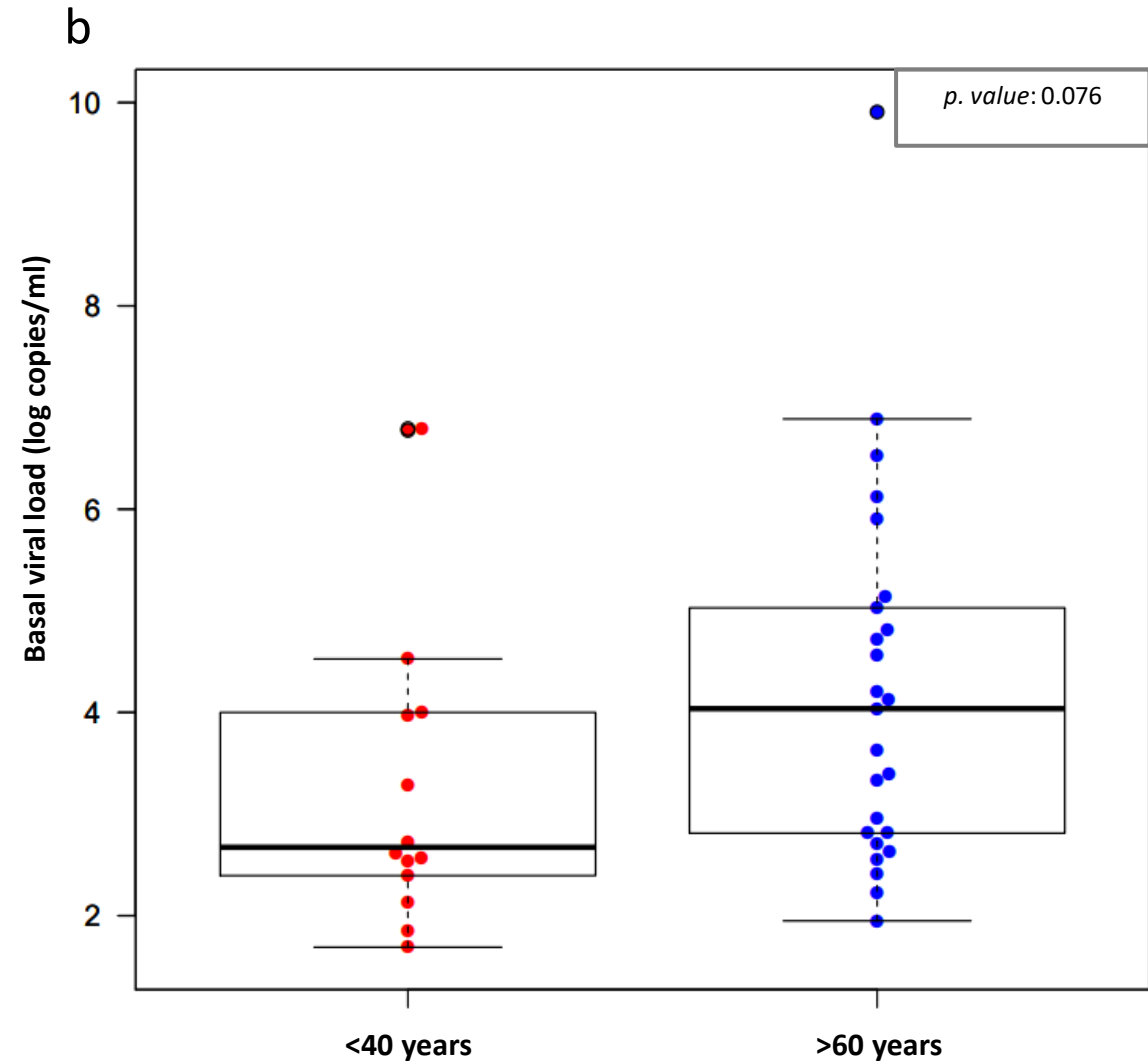

Supplement: Supplementary file 1 — Supplementary Figure 1. [file 41598_2021_3461_MOESM1_ESM.pdf]
