# Supplementary material for: Clinical evaluation of antiseptic mouth rinses to reduce salivary load of SARS-CoV-2
Source: Sci Rep. 2021 Dec 22;11:24392. doi: 10.1038/s41598-021-03461-y (PMC8695582; doi:10.1038/s41598-021-03461-y)
Supplement: Supplementary file 2 — Supplementary Table 1. [file 41598_2021_3461_MOESM2_ESM.pdf]

Supplementary Table 1. Individual salivary viral loads at each time point for the different mouthwash treatments (A: PVP-I, B: H_2O_2, C: CPC, D: CHX, E: Distilled water). The four columns correspond to the viral loads at baseline, and at 15, 30 and 60 minutes post-mouthwash.

Patients A	Log copies/ml	Log copies/ml	Log copies/ml	Log copies/ml
MAD 21-A1	5,90E+03	1,18E+05	2,74E+06	3,29E+05
MAD 81-A1	3,47E+02	2,69E+02	6,24E+01	1,07E+02
MAD 86-A1	3,63E+04	5,27E+05	5,71E+05	5,14E+04
MUR 61-A1	8,25E+06	8,89E+02	7,17E+04	1,52E+05
MUR 71-A1	6,56E+04	4,75E+04	1,89E+04	1,64E+04
MUR 76-A1	3,74E+02	2,78E+02	1,08E+04	2,61E+03
VLC 46-A1	5,22E+02	4,92E+02	0,00E+00	7,53E+01
VLC 46-A1 extra	1,37E+02	1,85E+02	0,00E+00	0,00E+00
VLC 51-A1 extra	1,70E+02	0,00E+00	8,24E+02	0,00E+00

Patients B	Log copies/ml	Log copies/ml	Log copies/ml	Log copies/ml
MAD 17-B1	3,94E+05	9,23E+04	8,07E+04	2,10E+04
MAD 22-B1	1,35E+06	3,82E+04	4,41E+04	8,54E+03
MAD 27-B1	8,76E+01	3,97E+03	0,00E+00	3,01E+03
MAD 32-B1	7,16E+03	8,34E+03	2,47E+04	1,32E+05
MAD 37-B1	2,12E+03	6,56E+01	1,09E+02	2,47E+02
MAD 82-B1	7,01E+01	9,18E+02	1,00E+02	1,21E+02
MAD 87-B1	1,28E+04	3,34E+05	1,22E+05	5,75E+03
MAD 92-B1	4,22E+02	3,46E+02	3,79E+01	0,00E+00
MUR 57-B1	7,57E+04	2,25E+04	5,90E+03	8,92E+05
MUR 62-B1	2,84E+03	1,25E+03	3,97E+03	2,67E+02
MUR 67-B1	1,07E+05	6,96E+07	1,67E+05	2,70E+05
MUR 72-B1	1,42E+02	1,67E+02	0,00E+00	1,72E+02
MUR 77-B1	2,53E+02	1,38E+02	7,97E+01	0,00E+00
VLC 52-B1	8,90E+01	7,18E+01	6,67E+02	1,11E+02

Patients C	Log copies/ml	Log copies/ml	Log copies/ml	Log copies/ml
MAD 23-C1	5,85E+02	7,03E+03	4,44E+02	2,58E+04
MAD 28-C1	2,52E+03	7,27E+03	1,08E+04	3,48E+05
MAD 33-C1	1,33E+04	1,54E+02	1,72E+06	3,46E+03
MAD 38-C1-ok	5,41E+02	1,77E+03	9,62E+03	2,47E+04
MAD 83-C1	7,91E+05	2,25E+04	5,22E+04	2,36E+02
MAD 88-C1	1,39E+05	2,91E+03	6,04E+03	5,52E+03
MUR 58-C1	3,38E+04	2,78E+04	3,15E+05	2,14E+03
MUR 63-C1	2,48E+02	0,00E+00	0,00E+00	0,00E+00
MUR 68-C1	2,17E+04	9,88E+03	1,16E+04	1,54E+04
MUR 78-C1	4,16E+03	4,78E+03	8,77E+03	1,26E+05
VLC 43-C1	6,60E+02	5,37E+03	0,00E+00	4,24E+03

Patients D	Log copies/ml	Log copies/ml	Log copies/ml	Log copies/ml
MAD 24-D1	3,42E+03	1,61E+03	1,30E+04	4,99E+04
MAD 29-D1	4,90E+01	4,25E+01	3,65E+01	0,00E+00
MAD 39-D1	1,17E+05	1,92E+05	8,13E+04	4,01E+03
MAD 84-D1	1,14E+03	2,24E+05	9,22E+03	1,35E+04
MAD 89-D1	7,02E+04	2,31E+05	2,68E+03	2,11E+03
MAD 94-D1	5,22E+04	1,76E+03	2,54E+02	7,90E+02
MUR 59-D1	1,95E+03	2,50E+02	2,89E+02	1,01E+02

MUR 69-D1	4,11E+02	1,66E+02	0,00E+00	5,15E+01
MUR 74-D1	4,05E+05	9,26E+05	1,26E+06	2,97E+07
VLC 44-D1	2,56E+02	2,45E+02	1,09E+03	2,88E+03
VLC 49-D1	3,59E+02	5,46E+04	1,88E+03	7,44E+03
VLC 54-D1	1,00E+04	7,05E+04	1,68E+04	2,44E+03

**Patients E**	**Log copies/ml**	**Log copies/ml**	**Log copies/ml**	**Log copies/ml**
MAD 20-E1	1,77E+02	3,27E+01	2,01E+04	4,26E+03
MAD 25-E1	9,47E+03	2,45E+05	0,00E+00	1,57E+03
MAD 30-E1	1,60E+04	1,16E+02	1,62E+02	1,25E+03
MAD 35-E1	0,00E+00	2,33E+02	4,20E+02	5,97E+01
MAD 40-E1	5,96E+06	1,94E+06	2,42E+07	6,85E+07
MAD 85-E1	3,36E+04	2,18E+04	2,64E+03	2,93E+03
MAD 90-E1	7,72E+06	1,11E+08	2,37E+07	5,94E+06
MAD 95-E1	8,09E+09	8,97E+07	7,95E+08	3,59E+03
MUR 16-E1-EXTRA	2,29E+06	1,67E+06	3,91E+05	2,93E+05
MUR 75-E1	1,09E+04	7,14E+05	1,41E+06	2,37E+02
MUR 80-E1	4,22E+03	3,67E+05	2,34E+05	1,12E+06
VLC 55-E1	7,21E+01	8,20E+01	4,28E+02	3,22E+02
