# Supplementary material for: Clinical evaluation of antiseptic mouth rinses to reduce salivary load of SARS-CoV-2
Source: Sci Rep. 2021 Dec 22;11:24392. doi: 10.1038/s41598-021-03461-y (PMC8695582; doi:10.1038/s41598-021-03461-y)
Supplement: Supplementary file 3 — Supplementary Legends. [file 41598_2021_3461_MOESM3_ESM.docx]

**Supplementary Figure 1. Relationship between patient age and salivary viral load.** Spearman's correlation coefficient between basal viral load and age is displayed on panel a. Box plots in b show levels of viral load at baseline in patients under 40 years of age (in red) and in patients over 60 years of age (in blue). Wilcox test result is also indicated.
